# Volume kinetic analysis of fluid retention after induction of general anesthesia

**DOI:** 10.1186/s12871-020-01001-1

**Published:** 2020-04-25

**Authors:** Robert G. Hahn, Janis Nemme

**Affiliations:** 1 Research Unit, Södertälje Hospital, 152 86 Södertälje, Sweden; 2 Karolinska Institutet at Danderyds Hospital (KIDS), Stockholm, Sweden; 3 Department of Anesthesiology and Intensive Care, Riga Stradins University, Riga, Latvia; 4 Paul Stradins Clinical University Hospital, Riga, Latvia

**Keywords:** Ringer’s lactate, Brain natriuretic peptide, Heparan sulfate, Syndecan-1, Pharmacokinetics

## Abstract

**Background:**

Induction of general anesthesia increases the hemodilution resulting from infusion of crystalloid fluid, which is believed to be due to slower distribution caused by arterial hypotension. When normal distribution returns is not known.

**Methods:**

An intravenous infusion of 25 mL kg^− 1^ of Ringer’s lactate was infused over 30 min to 25 volunteers just after induction of general anesthesia for open abdominal hysterectomy. A two-volume model was fitted to the repeated measurements of the blood hemoglobin concentration and the urinary excretion using mixed-effects modelling software. Individual-specific covariates were added in sequence.

**Results:**

Distribution of infused fluid was interrupted during the first 20 min of the infusions. During this time 16.6 mL kg^− 1^ of lactated Ringer’s had been infused, of which virtually all remained in the circulating blood. Thereafter, the fluid kinetics was similar to that previously been found in awake volunteers except for the elimination rate constant (*k*_10_), which remained to be very low (0.86 × 10^− 3^ min^− 1^). Redistribution of infused fluid from the interstitium to the plasma occurred faster (higher *k*_21_) when the arterial pressure was low. No covariance was found between the fixed parameters and preoperatively concentrated urine, the use of sevoflurane or propofol to maintain the anesthesia, or the plasma concentrations of two degradation products of the endothelial glycocalyx, syndecan-1 and heparan sulfate.

**Conclusions:**

Induction of general anesthesia interrupted the distribution of lactated Ringer’s solution up to when 16.6 mL kg^− 1^ of crystalloid fluid had been infused. Plasma volume expansion during this period of time was pronounced.

**Trial registration:**

Controlled-trials.com (ISRCTN81005631) on May 17, 2016 (retrospectively registered).

## Background

Pharmacokinetic methods can be applied to fluid volumes, which is of interest in anesthesia and surgery where large amounts are given by intravenous infusion. The most commonly used approach is volume kinetics, which uses the hemodilution and the urinary excretion as input in the calculations [1, 2]. The hemodilution is the inverse of the blood water concentration and, therefore, shows how the infused volume is distributed; as per volume, the blood contains little more than hemoglobin and water [2, 3]. So far, volume kinetics has dealt with anesthesia and surgery where fluid is given to ensure adequate organ perfusion. The volume of infused fluid is critical because the elimination efficacy is strongly impaired during general anesthesia [4–6]. However, certain issues still remain to be resolved with regard to the kinetics of crystalloid fluid in this setting.

One such issue is why excessive hemodilution from infused fluid develops when induction of general epidural, spinal or anesthesia is associated with hypotension [7–10] but not when the arterial pressure is unchanged [7, 11]. A reasonable interpretation of this relationship is that anesthesia-induced vasodilatation lowers the arterial pressure and, in turn, increases the intravascular retention of infused fluid by reducing the capillary filtration. However, the exchange of fluid between the plasma to the interstitial fluid space occurs at a fairly normal rate during ongoing surgery even when the arterial pressure is low [12]. Molecular mechanisms, such as a rise of the plasma concentrations of brain natriuretic peptide (BNP) and glycocalyx degradation products [13–15], may also have a role in this process.

Another issue was to examine whether the urinary concentration of metabolic waste products before the operation reduces rate of subsequent elimination of infused fluid, which has been shown in conscious patients [16, 17]. For this purpose, fluid volume kinetic analysis was performed on a crystalloid fluid load of 25 mL kg^− 1^ given before open hysterectomy.

The primary hypothesis was that the excessive intravascular fluid retention after induction of general anesthesia is caused by a reduction of the capillary leakage, as given by the kinetic model. The secondary hypothesis was that a mathematical link exists between volume kinetic parameters and the plasma concentrations of BNP, glycocalyx degradation products and the urinary concentration of metabolic waste products. The kinetic analysis served as primary outcome measure and the measurements of metabolic waste products and their associations with the kinetics as secondary outcome measures.

## Methods

A total of 25 clinical patients scheduled for elective abdominal hysterectomy were recruited to an open label randomized parallel clinical trial with the primary aim to compare two anesthesia methods, sevoflurane and intravenous propofol, with regard to their influence the degradation of the endothelial glycocalyx layer [18]. Ethical approval (No. 270116-17 L) was provided by the Ethics Committee of Riga Stradins University (Chairperson P. Stradins) on January 27, 2016, and registered at controlled-trials.com as ISRCTN81005631 on May 17, 2016 (retrospectively, first patient studied on March 22, 2016). All patients gave us their written informed consent to participate. Patients were included if being aged 25–55 years, free from cardiopulmonary or renal disease, and complicated surgery was not expected. The study adheres to the CONSORT Guidelines. After fasting overnight, patients were given general anesthesia induced with propofol but maintained with either sevoflurane as inhaled anesthetic (*N* = 13) or an intravenous propofol infusion (*N* = 12). The result of that comparison is described elsewhere [18].

### Volume loading, blood sampling, and analysis

At the start of surgery, an intravenous infusion of Ringer’s lactate (25 mL kg^− 1^) was administered over 30 min. No more fluids were given except for the anesthesia drugs. No vasopressor or inotrophic agent was used. Blood (3 mL) was taken from a cubital vein for measurement of the blood hemoglobin (Hb) concentration on a Coulter HMx 5-diff (Beckman Coulter Inc., Brea, CA) with a coefficient of variation (CV) of 0.7% was taken every 10 min during the 30-min infusions and then every 15 min up to the end of surgery.

Blood (3 mL) and urine were also sampled at baseline, after anesthesia induction, and then at 30, 60, and 90 min later after the fluid infusion had been initiated, and 2 h after the anesthesia had been terminated. These samples were used to measure the plasma concentration of brain natriuretic peptide (BNP) on an Architect i2000, Abbott Park, Illinois, USA with a CV of 5.6%. The normal range is < 100 ng L^− 1^. Results below the limit of detection (10 ng L^− 1^) were set to that value. Moreover, the plasma concentrations of two endothelial shedding products, syndecan-1 and heparan sulfate, were analyzed using enzyme-linked immunosorbent assay (ELISA) kits from Diaclone, France, and Amsbio, Abingdon, UK, with CV% values of 6.2, and < 10%, respectively. The normal value of syndecan-1 for humans is 32 ng/mL (manufacturer data) and for heparan sulfate it is 5.9 μg mL^− 1^ [14].

Urinary creatinine concentration was measured within 36 h on a Cobas Integra 400 Plus instrument (Roche Diagnostics, Switzerland) with a CV of 2%. The urine osmolality was measured with an Osmomat 3000 (Gonotech, Berlin, Germany) and the urine-specific weight on a Urisys 2400 (Roche Diagnostics, Switzerland) with CVs being 3 and 0.1%, respectively.

### Population volume kinetics

Volume kinetics is a method for analyzing the distribution and elimination of infusion fluids [1, 2]. The approach has similarities to drug pharmacokinetics but uses the excreted urine volume and the plasma dilution derived from serial analyses of the blood Hb concentration as inputs in the calculations.

**Fig. 1:**
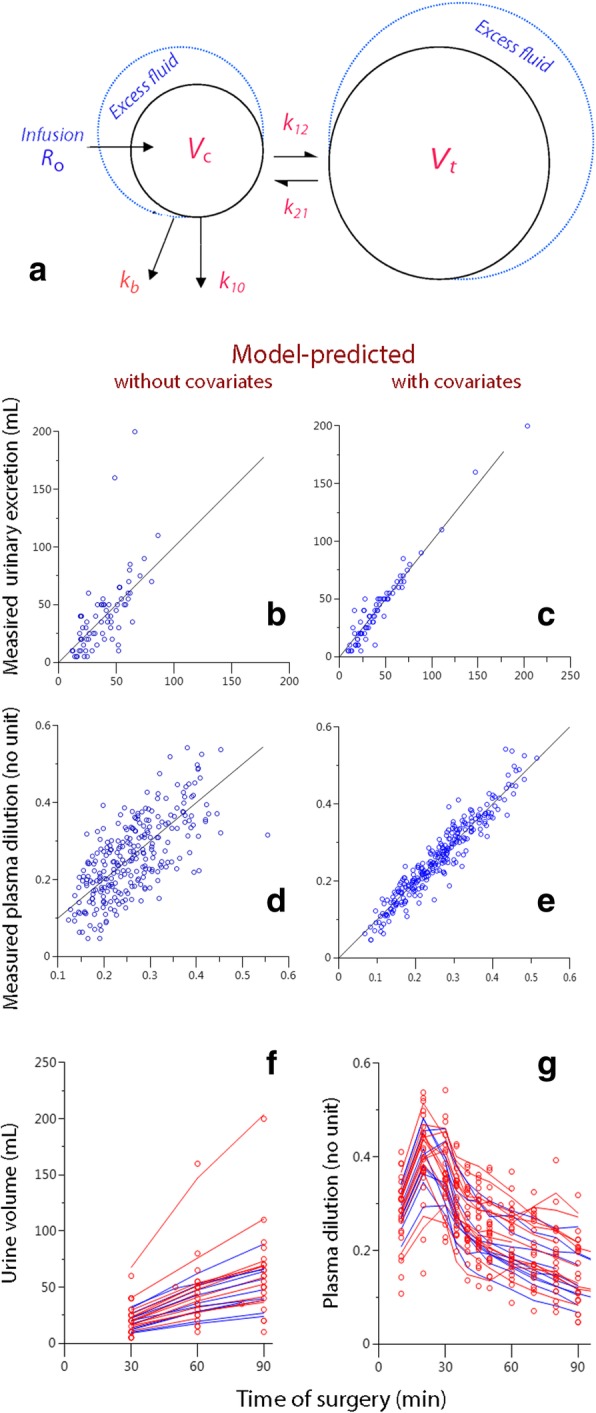
The kinetic model (a), residual plots (b-e) and final curve fits (f and g)

All included patients are analyzed in a single run, and then the influence of various covariates on the model parameters is tested sequentially. The model is built to agree with physiological data where isotonic infusion fluids distribute between two compartments: the plasma and the interstitial fluid space. Several models have been attempted to characterize fluid kinetics during surgery, but the one shown in Fig. 1a has been found to be most appropriate: fluid is infused into an expandable central (*V*_c_) space and becomes distributed to the peripheral (*V*_t_) fluid space. Fluid is translocated from *V*_c_ to *V*_t_ in proportion by a rate constant *k*_12_ to the central volume expansion, which is written as (*v*_c_ – *V*_c_). Fluid is returned from *V*_t_ to *V*_c_ via another rate constant, *k*_21_. Elimination occurs from *V*_c_ by one or two routes (*k*_10_ and *k*_b_) in proportion to (*v*_c_ – *V*_c_). The rate constant *k*_10_ represents fluid that is collected as urine while *k*_b_ governs fluid filtered from *V*_c_ that does not equilibrate with *V*_c_ or *V*_t_ during the period of the study [5, 6, 19, 20].

The differential equations for the single-elimination route model are:

1 $$ {dv}_c/ dt={R}_o-{k}_{12}\left({v}_c-{V}_c\right)+{k}_{21}\left({v}_t-{V}_t\right)-{k}_{10}\left({v}_c-{V}_c\right)-{k}_b\left({v}_c-{V}_c\right) $$

2 $$ {dv}_t/ dt={k}_{12}\left({v}_c-{V}_c\right)-{k}_{21}\left({v}_t-{V}_t\right) $$

where *R*_o_ is the rate of infusion, *v*_c_ and *v*_t_ are the constantly changing volumes of distribution for newly infused fluid, and *V*_c_ and *V*_t_ are the baseline volumes.

The Hb-derived fractional plasma dilution was used to indicate the volume expansion of *V*_c_ resulting from the infusion. Hence:

3 $$ \left({v}_c-{V}_c\right)/{V}_c=\left[\left(\mathrm{Hb}/\mathrm{hb}\right)-1\right)\Big]/\left(1-\mathrm{baselinehematocrit}\right) $$

Symbols in capital letters denote baseline values. A minor correction was made for the effects of surgical hemorrhage and blood sampling on the plasma dilution [1, 12]. The measured urinary excretion during 30-min intervals was then used to estimate *k*_10_ as follows (AUC = area under the curve):

4 $$ {k}_{10}=\mathrm{urinaryexcretion}/\mathrm{AUC}\mathrm{for}\left({v}_c-{V}_c\right) $$

### Covariate analysis

Twenty individual-specific covariates were evaluated in a forward stepwise fashion, as guided by plots of random effects (“eta” plots). The routine for identifying statistically justified covariates is described in detail elsewhere [4]. In short, a covariate was included if it decreased the residual error of the model (expressed as − 2 log likelihood by 3.8, which corresponds to *P* < 0.05), the 95% confidence interval of the parameter estimate did not include zero, and the CV% for the inter-individual variability was < 50%.

Continuous covariates assessed only once were age, body weight, surgical blood loss, C-reactive protein, the use of sevoflurane or propofol for maintaining the anesthesia, the syndecan-1 and heparan sulfate concentrations before and 2 h after the surgery, and the preoperative urinary specific gravity, osmolality, and the urinary creatinine concentration. The plasma concentrations of syndecan-1 and heparan sulfate served as time-varying covariates and were included four times per operation, while heart rate and the systolic, diastolic, and mean arterial pressure (MAP) were assessed at every blood sampling time point. The time period before and after 10, 20, and 30 min were evaluated as categorical covariates in an exponential covariate model, as fluid was infused at a rate of 0.833 mL kg^− 1^ min^− 1^ and our belief was that the fluid kinetics would change depending on the infused volume. In the last situation, all time points up to 30 min were set to 1 and all time points after 30 min were given the value 0.

The kinetic program used was Phoenix software for nonlinear mixed effects (NLME), version 1.3 (Certara, St. Louis, MO). The goodness-of-fit of the model was illustrated by residual plots and the performance of the model by predictive checks.

The study was powered to detect a doubling of the plasma BNP concentration as a result of the volume loading, as described previously [18]. An effect size of 1.0 and power 90% at the *P* < 0.01 level yielded *N* = 20. Data with a normal distribution are presented as the mean (SD), data with a skewed distribution as the median (25th–75th percentiles) and kinetic output as the mean (CI). *P* < 0.05 was statistically significant.

## Results

### Kinetic analysis of Hb changes

The kinetic analysis was based on 298 time points during the 25 operations. All data are given in the Additonal file 1 and the model used is illustrated in Fig. 1a. Selected key characteristics of the operations are shown in Table 1.

**Table 1:** Key surgical variables

Measured Variable	Result
N	25
Age (years)	47 (5)
Body weight (kg)	75 (14)
Infused fluid volume (mL)	1869 (336)
Mean arterial pressure (mmHg)	
Before induction	100 (15)
After induction	80 (10)
End of infusion	92 (17)
During surgery^a^	85 (14)
End of study (90 min)	91 (18)
Blood Hb (g/L)	
Before induction	119.5 (16.3)
After induction	117.1 (15.9)
End of infusion	95.8 (14.2)
End of study (90 min)	104.6 (15.8)
Brain natriuretic peptide (BNP; ng/L)^a^	20.9 (11.2)
Plasma syndecan-1 (ng/mL)^a^	12.8 (8.6–20.9)
Plasma heparan sulfate^a^	6.5 (4.9–10.3)
Operating time (min)	91 (10)
Blood loss (mL)	150 (100–162)
Urinary excretion (mL)	50 (39–70)

^a^ based on the mean for all intraoperative measurements

Data are mean (SD) or median (25th–75th percentile)

The search strategy used to find the optimal parameter estimates is shown in Table 2. The final base model with the fixed model parameters is shown in the upper part of Table 3 and the covariate effects in the lower part of Table 3. Two routes of elimination were optimal; one of these (*k*_10_) corresponded to the measured urinary excretion.

**Table 2:** Key features of the search protocol used to find the final population kinetic model

Optimization routine	Added covariate	Target parameter	LL	-2(LL)	AIC
Naive			−72	152	165
FOCE LB			57	−113	−93
+ 2 elimination routes			67	−134	−110
	Time ≤ 20 min	*k*_12_	111	−219	−193
	Time ≤ 20 min	*k*_*b*_	113	−227	−200
	Time ≤ 20 min	*V*_c_	128	−256	−227
	Body weight	*V*_c_	140	−280	−248
	BNP	*k*_10_	143	−285	−251
FOCE ELS	MAP	*k*_21_	159	−318	−282

A reduction −2(LL) by 3.8 is significant by *P* < 0.05 and > 6.6 points by *P* < 0.01)

*FOCE LB* First-Order Conditional Estimation according to Lindstrom-Bates, *FOCE ELS* FOCE Extended Least Squares, *LL* Log likelihood, *AIC* Akaike Criterion

**Table 3:** Population kinetic parameters in the final model

	Covariate	Best estimate	2.5% CI	97.5% CI	CV%
Fixed parameter					
tvV_c_ (mL)	–	3205	2906	3504	4.8
tv*k*_12_ (10^− 3^ min^− 1^)	–	69.0	46.3	91.7	16.7
tv*k*_21_ (10^− 3^ min^−1^)	–	93.5	67.8	119.5	14.1
tv*k*_10_ (10^− 3^ min^−1^)	–	0.86	0.72	1.00	8.4
tv*k*_b_ (10^− 3^ min^−1^)	–	15.8	12.0	19.6	12.1
Covariate effect					
*k*_12_	Time ≤ 20 min	−16.5	− 19.0	−14.0	−7.8
*k*_b_	Time ≤ 20 min	− 16.5	−7.9	−19.0	− 7.9
*V*_c_	Time ≤ 20 min	−0.35	− 0.44	− 0.26	− 13.3
*V*_c_	Body weight	1.49	0.96	2.03	18.2
*k*_10_	BNP	0.37	0.12	0.62	33.9
*k*_21_	MAP	−1.47	−1.99	−0.94	− 18.1

The three “Time” covariates are exponential models, the others are power models

*BNP* Brain natriuretic peptide, *MAP* Mean arterial pressure, *tv* Typical value, *CI* Confidence interval, *CV* Coefficient of variation (inter-individual)

Comparisons between the measured and predicted urinary excretion and the plasma dilution based on the fixed parameters alone are shown in Fig. 1b and c. The corresponding plots that also consider the covariates are presented in Fig. 1d and e, and the final curve fits for each individual are displayed in Fig. 1f and h.

### Predictive check and covariates

**Fig. 2:**
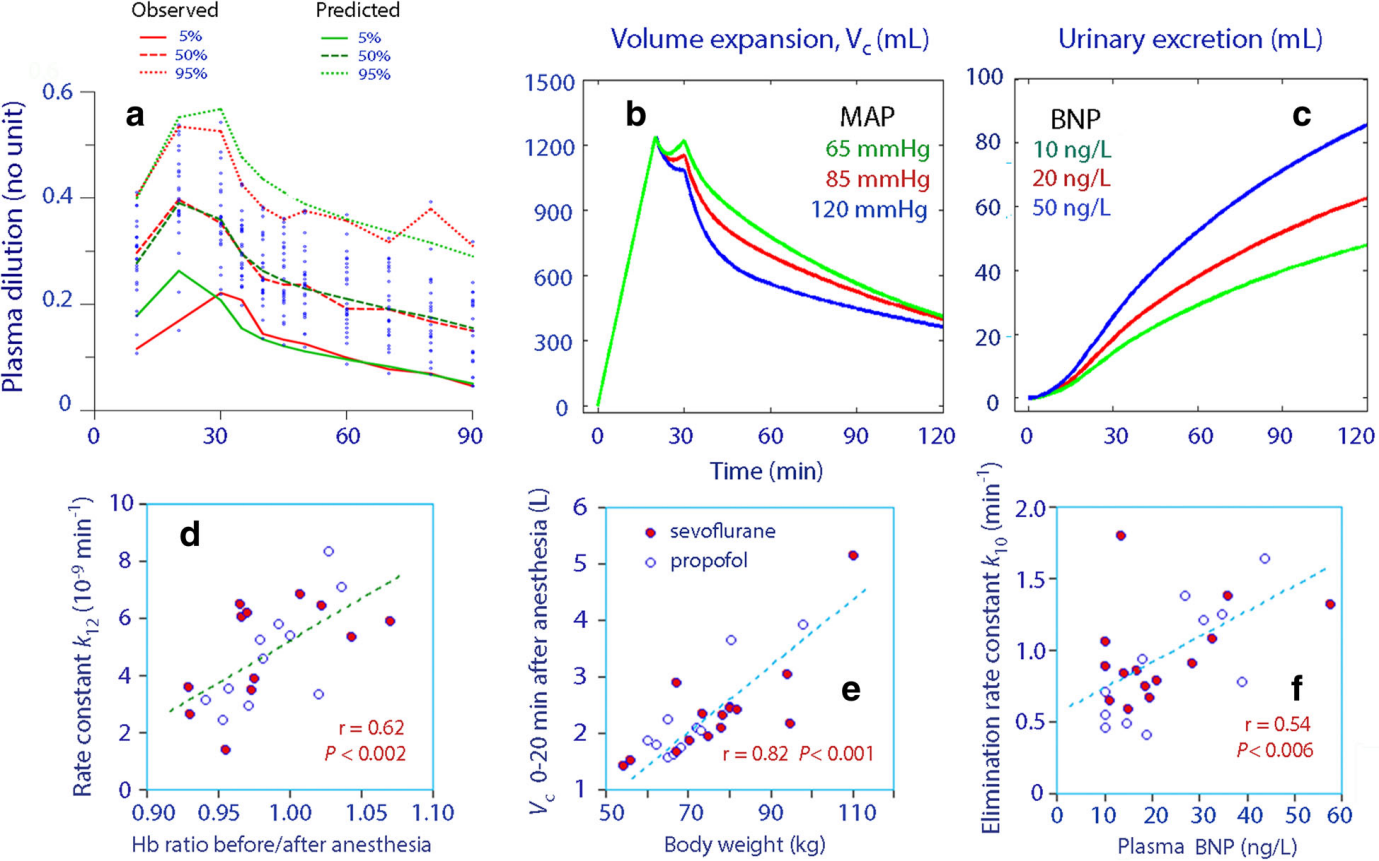
Predictive check of the kinetic analysis (a). Computer-based simulations (b and c). Correlations between measured parameters and kinetic constants (d, e and f)

A predictive check the kinetic model, based on 1000 simulations, is given in Fig. 2a. The close agreement between the percentiles for the predicted and the observed data indicates good model performance and that the model is robust.

The most important covariate was “Time ≤ 20 min,” which means that *k*_12_, *k*_b_, and *V*_c_ showed lower values during the first 20 min of the infusion, when 2/3 of 25 mL kg^− 1^ = 16.6 mL kg^− 1^ (mean 1245 mL) of lactated Ringer’s was infused. An exponential covariate model was used to examine if a more precise prediction could be obtained by placing the cut-off at other points in time, but the fluid volume given up to 20 min was found to be the optimal. The strong inhibition of leakage of fluid from the plasma allowed the infusion to raise the plasma volume more rapidly. Consequently, the plasma volume reached a maximum by 20 min (instead of 30 min) (Fig. 2b).

A low MAP increased the plasma volume expansion by virtue of a negative covariance with *k*_21_ (power model with MAP overall mean 85 mmHg; Fig. 2b).

A high BNP increased the urinary excretion over time (Fig. 2c).

The lowest values for *k*_12_ were obtained in patients who had developed spontaneous hemodilution during the induction of anesthesia (Fig. 2d).

*V*_c_ also increased with the body weight (power model, mean 74.8 kg; Fig. 2e) and *k*_10_ with the plasma concentration of BNP (power model; mean 20.9 ng L^− 1^; Fig. 2f).

Factors that did not serve as statistically significant covariates to any of the fixed parameters included age, the preoperative biomarkers of concentrated urine, the use of sevoflurane or propofol, and the plasma concentrations of levels of C-reactive protein and the two shedding substances that reflect glycocalyx degradation.

Additonal file 2 is a list of all potential covariates and their associations with the fixed parameters.

### Concentrated urine

**Fig. 3:**
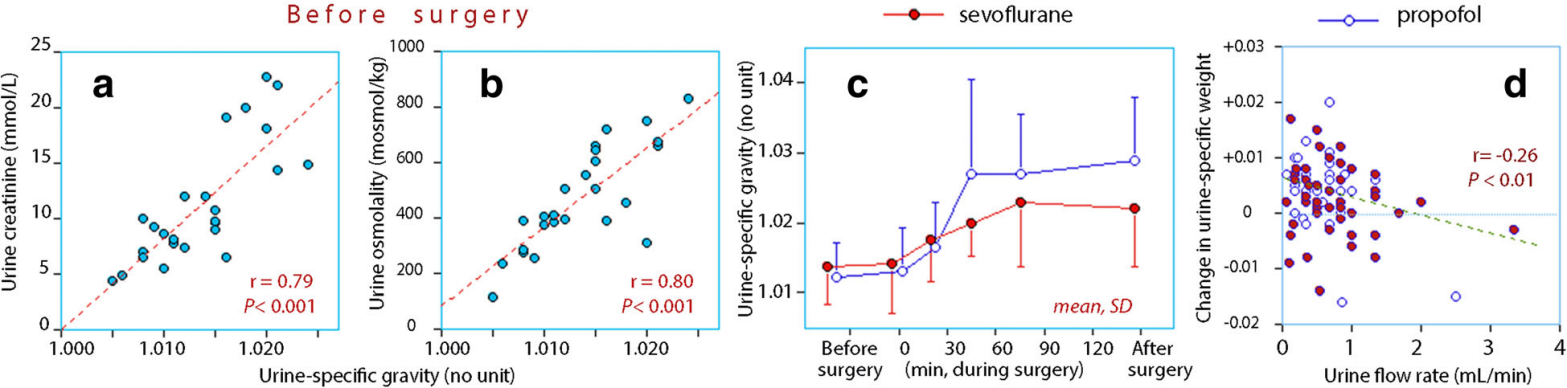
Concentrated urine. One extreme outlier was omitted in the subplot in D, which shows the change in urine-specific weight over a period of 30 min versus the urine flow rate

The three markers of concentrated urine (urine-specific gravity, creatinine, and osmolality) correlated closely (Fig. 3a and b). None of them served as statistically significant covariate to the fluid kinetics.

The urine became more concentrated during the surgery (*P* < 0.001 repeated-measures ANOVA) and remained most concentrated in the propofol group at 2 h (*P* < 0.04; Fig. 3c). The overall low urine flow rate promoted further concentration, particularly if the urine flow was less than 1 mL min^− 1^ during 30 min (Fig. 3d).

## Discussion

The pharmacokinetic approach used here, volume kinetics, uses the inverse of the hemodilution and the urinary excretion as input in pharmacokinetic calculations [1, 2]. The calculations distinguish a “wall” between a central (*V*_c_) and an extravascular compartment (*V*_t_), which probably represents the difficulty for fluid to expand the interstitial fluid space. The hemodilution pattern, and not the magnitude of the hemodilution, determines how much fluid is allocated to each one of the two spaces. In the present study, the *V*_c_ volume was 3.2 L, which is the volume with which the infused fluid quickly equilibrates. It also corresponds closely to the expected plasma volume.

The present study shows that induction of general anesthesia transiently changes the kinetics of infused crystalloid fluid. The most striking finding was a strong inhibition of the distribution of fluid during the first 20 min of the infusion, up to when 16.6 mL kg^− 1^ body weight had been infused. The rate constant *k*_12_ was then reduced to a small fraction of the normal value found during the remainder of the experiment. Figure 2b illustrates that the amount of fluid residing in the central compartment *V*_c_ at 20 min closely agrees with the total amount of fluid that had been infused at that time. The anesthesia-induced vasodilatation and the resulting drop in the MAP by 20 mmHg are sufficient to explain the almost complete “shut-off” of the distribution [4]. However, the present study shows, for the first time, when fluid filtration begins again, and that is when approximately 1.25 L of fluid has been infused and the plasma volume has increased by the same volume.

The results would be quite different if anesthesia had not been induced. The hypotension-associated interruption of capillary filtration doubled the maximum plasma dilution in response to 25 mL kg^− 1^ of crystalloid fluid from the expected 20% [21, 22] to 40% (Fig. 1 g and Fig. 2 a). In the absence of hemorrhage, these figures can the transposed into the relative (%) increase of the plasma volume.

Spontaneous hemodilution, which averaged 2% but occasionally reached 7–8%, occurred during the induction of anesthesia. i.e. before any fluid had been infused, which degree correlated with the very low *k*_12_ that prevailed until 16.6 mL kg^− 1^ of lactated Ringer’s had been infused (Fig. 2d). There was also a temporarily smaller *V*_c_, which suggests that the fluid infused early primarily became enriched in the central circulation. The restoration of *V*_c_ and *k*_12_ after 16.6 mL kg^− 1^ had been infused accounts for the unexpected observation that the greatest plasma volume expansion occurred before the end of the infusion.

The diuretic response to infused fluid is reduced by approximately 90% during general anesthesia, which is strongly associated with the reduction of MAP [4–7]. In the present study, all patients underwent general anesthesia, but covariance between MAP and *k*_10_ was difficult to demonstrate due to the very low overall *k*_10_; the parameter value was only 0.00086 (i.e. half-life of 13 h) while the corresponding value in cohorts of conscious volunteers has varied between 0.015 and 0.028 (half-life 25–50 min) [19, 20]. Interestingly, most of the infused fluid was not eliminated as urine but in proportion by the rate constant *k*_b_ to the expansion of *V*_c_ at any given time. The fluid that was eliminated from the kinetic system by this mechanism remained in the body but did not equilibrate with the plasma during the period of the study. A higher *k*_b_ than *k*_10_ has previously been reported only during general anesthesia [2, 23, 24].

The poor diuretic response to infused fluid might also explain the lack of covariance between fluid kinetics and concentrated urine, which is index of low habitual intake of water and/or overt dehydration [25, 26]. An inhibitory effect of preoperatively concentrated urine on the diuretic response to volume loading with crystalloid fluid [16] and 20% albumin [17] has previously been found in conscious patients. In the present study, the process of further concentrating the urine during the surgery was followed in a step-wise fashion. The urine became gradually more concentrated (Fig. 3c) and was most likely to occur whenever the urine flow rate fell below 1 mL min^− 1^ (Fig. 3c). The theoretical importance of this process of is that patients with a low habitual intake of water might have their urine so much more concentrated during the surgery that the concentrating capacity of the kidneys is exceeded, which causes a postoperative rise in plasma creatinine [27–29]. Unfortunately, plasma creatinine was not measured after the surgery.

Although the diuresis was very small, an elevated BNP was associated with slightly increased urine volumes (Fig. 2c, f). This biomarker of ventricular distention doubles in response to volume loading with 25 mL min^− 1^ of crystalloid fluid in awake volunteers [30] but apparently to a smaller degree during general anesthesia.

A currently popular view is that surgical trauma and hypervolemia cause degradation of the endothelial glycocalyx layer whereby the capillary leakage of macromolecules and fluid would increase [13–15]. The degree of acute glycocalyx degradation can be evaluated by measuring the plasma concentrations of, for example, syndecan-1 and heparan sulfate. The present data did not disclose any marked elevations of these biomarkers (Table 1). Their plasma concentrations actually followed the hemodilution pattern quite well and showed no covariance with *k*_12_ or *k*_b_, which are else the relevant parameters to indicate increased capillary leakage.

Limitations include that capillary filtration was regained only after having administrated a fairly large bolus volume of Ringer’s. When a Starling equilibrium adequate for general anesthesia has developed after infusion of smaller amounts of fluid, such as 500 mL, is unknown. Slow capillary refill of the same kind as during induction of anesthesia would probably continue but, in any event, equilibrium would certainly occur later than the 20 min found here.

The cut-off points for changed fluid kinetics were evaluated only at 10-min intervals. A higher resolution could have been obtained by sampling blood at shorter intervals. Not only the infused fluid volume, but also the time from the anesthesia induction, might contribute to restoration of the distribution function. No massive elevations of the biomarkers for cardiac strain and glycocalyx degradation were found, which limits the possibilities to identify them as covariates. Finally, the kinetic model is constructed to reasonably well agree with known body physiology, but what the fixed kinetic parameters represent is still not finally proven.

## Conclusions

A fluid volume kinetic method shows the excessive hemodilution occurring when crystalloid fluid is infused after induction of general anesthesia corresponds to a nearly complete interruption of the capillary filtration up to the point in time when 16.6 mL kg^− 1^ of lactated Ringer’s has been infused. The small urinary excretion did not cause intravascular overload as fluid could be filtered to extravascular tissues both with and without being in equilibrium with the plasma.

## Supplementary information

**Additional file 1.**

**Additional file 2.**

## Data Availability

All data available as a Additonal file 1 and all covariances as Additonal file 2.

## Abbreviations

ANOVA: Analysis of variance
BNP: Brain natriuretic peptide
AUC: Area under the curve
Hb: Hemoglobin
LL: Log likelihood
h: Hour
L: Liter
mL: Milliliter
*k*_12_: Rate constant for fluid passing from *v*_c_ to *v*_t_
*k*_21_: Rate constant for fluid passing from *v*_t_ to *v*_c_
*k*_10_: Rate constant for fluid leaving the kinetic system as urine
*k*_b_: Rate constant for fluid leaving the kinetic system but not as urine
MAP: Mean arterial pressure
R_o_: Rate of infusion
*V*_c_ and *v*_c_: Size of central body fluid space at baseline and during fluid therapy, respectively
*V*_t_ and *v*_t_: Size of peripheral body fluid space at baseline and during fluid therapy, respectively
